# A novel MnO–CrN nanocomposite based non-enzymatic hydrogen peroxide sensor†

**DOI:** 10.1039/d1ra01485d

**Published:** 2021-05-27

**Authors:** Ayesha Khan Tareen, Karim Khan, Waqas Ahmad, Muhammad Farooq Khan, Qudrat Ullah Khan, Xinke Liu

**Affiliations:** College of Materials Science and Engineering, Shenzhen University Nanhai Ave 3688 Shenzhen Guangdong 518060 P. R. China xkliu@szu.edu.cn; Institute of Microscale Optoelectronics, Collaborative Innovation Centre for Optoelectronic Science & Technology, Key Laboratory of Optoelectronic Devices and Systems of Ministry of Education and Guangdong Province, College of Physics and Optoelectronic Engineering, Shenzhen Key Laboratory of Micro-Nano Photonic Information Technology, Guangdong Laboratory of Artificial Intelligence and Digital Economy (SZ), Shenzhen University Shenzhen 518060 P. R. China karim_khan_niazi@yahoo.com; School of Electrical Engineering & Intelligentization, Dongguan University of Technology, Dongguan (DGUT) Dongguan Guangdong Province 523808 P. R. China; International Collaborative Laboratory of 2D Materials for Optoelectronics Science and Technology of Ministry of Education, Institute of Microscale Optoelectronics, Shenzhen University Shenzhen 518060 P. R. China; Department of Electrical Engineering, Sejong University 209 Neungdong-ro Gwangjin-gu 05006 South Korea; Key Laboratory of Optoelectronic Devices and Systems, Ministry of Education and Guangdong Province, College of Physics and Optoelectronics Engineering, Shenzhen University Shenzhen 518060 China

## Abstract

A MnO–CrN composite was obtained *via* the ammonolysis of the low-cost nitride precursors Cr(NO_3_)_3_·9H_2_O and Mn(NO_3_)_2_·4H_2_O at 800 °C for 8 h using a sol–gel method. The specific surface area of the synthesized powder was measured *via* BET analysis and it was found to be 262 m^2^ g^−1^. Regarding its application, the electrochemical sensing performance toward hydrogen peroxide (H_2_O_2_) was studied *via* applying cyclic voltammetry (CV) and amperometry (*i*–*t*) analysis. The linear response range was 0.33–15 000 μM with a correlation coefficient (*R*^2^) value of 0.995. Excellent performance toward H_2_O_2_ was observed with a limit of detection of 0.059 μM, a limit of quantification of 0.199 μM, and sensitivity of 2156.25 μA mM^−1^ cm^−2^. A short response time of within 2 s was achieved. Hence, we develop and offer an efficient approach for synthesizing a new cost-efficient material for H_2_O_2_ sensing.

## Abstract 2

A MnO–CrN composite was obtained *via* the ammonolysis of the low-cost nitride precursors Cr(NO_3_)_3_·9H_2_O and Mn(NO_3_)_2_·4H_2_O at 800 °C for 8 h using a sol–gel method.

## Graphical abstract

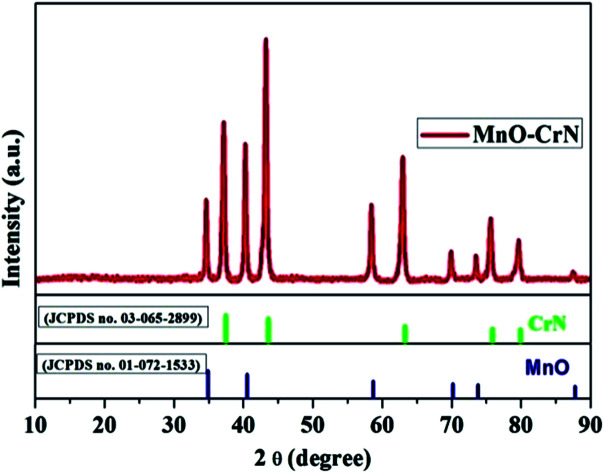

## Introduction

Hydrogen peroxide (H_2_O_2_) plays two important roles in living bodies. On one hand, it is involved in essential processes, like cell signaling and fighting microbial trespassers. On the other hand, it is associated with malfunction, ageing, and even cell death.^1,2^ In the reactive oxygen species family, H_2_O_2_ is an important member, and it is considered the most long-lived of the more reactive oxygen species (living longer than hydroxyl radicals and superoxide anions, for instance). It accumulates inside and outside of cells, where it is involved in both vital (signaling) and deadly (toxic) reactions depending on its concentration^3–5^. As a reactive oxygen species, H_2_O_2_ is involved in a variety of redox reactions within the living body.^6,7^ From kinetics studies, the most common range for H_2_O_2_ in healthy human plasma is 1–5 μM.^8^ Maintaining reactive oxygen species levels at or below this certain threshold concentration is essential for cellular endurance.^2^ The quantification of reactive oxygen species concentrations remains challenging and it is frequently not accomplished in biomedical studies due to a lack of suitable methods. To quantify these levels, electrochemical-based enzymatic/non-enzymatic sensors have been developed.^9,10^

Electrochemical biosensors based on the enzymatic catalysis of a reaction produce or consume electrons (redox enzymes). They work *via* direct electron transfer from proteins or the redox states of connected species that are changed in response to intermediate redox counterparts in protein prosthetic groups or appropriate co-substrates at active sites^11^. Enzymatic sensors have quick response times but they are very costly and only have low detection limits close to neutral pH, where they also have limited stabilities^12^. Biosensors also cannot be used for tracing extracellularly released H_2_O_2_ in cells because of the inadequate electron transfer activity, and the reactivity of immobilized enzymes is poor due to the lack of direction of immobilized enzymes on the electrode surface. Low reproducibility and insufficient stability have restricted the broad use of enzymatic sensors. Hence, non-enzymatic electrochemical H_2_O_2_ sensors are desirable.

Other than applications in living organisms, non-enzymatic sensors are also essential in the clinical,^13^ medical,^14^ food,^15^ packaging, and waste treatment fields. Therefore, developing a selective, rapid, and accurate H_2_O_2_ sensor with a wide detection range is of great interest to the environmental monitoring, pharmaceutical, and food industries. Measuring H_2_O_2_ with extremely high sensitivity usually requires horseradish peroxidase (HRP), mediators, or precious metal nanoparticles, resulting in the problems of low long-term operational stability and rising cost. Therefore, new materials are required for non-enzymatic sensors.

Recently, manganese-oxide compounds have received special attention on account of their prominent advantages, as they are low-cost, earth-abundant, and environmentally friendly, and they show considerable ORR activity. Generally, MnO_2_ is a semiconductor material,^16^ so we combined it with CrN as a conduction support material and synthesized a new material at the nano-level *via* a new facile synthesis method. It was established that the catalytic activity of the MnO–CrN composite surface is stimulated in the presence of hetero atoms (N, O, or others).^17^ Moreover, structural substitution is a well-known reactive approach for enhancing conductivity and increasing the functionality of materials, mostly with mixed valence species (*e.g.*, MnO_*x*_, CrO_*x*_, *etc.*). Redox reactions between Cr^3+^ and Mn oxidants were designed to control structural substitution *via* the use of a narrow onset potential range. We prepared hierarchical nitrogen-implanted MnO–CrN composite nanoparticles. The advantage of electrochemical sensors based on nanoparticles is that they can decrease the over-potentials of many analytes compared with unmodified electrodes.^18–20^ The novelty of this work is that we demonstrated, for the first time, MnO–CrN_2_ nanoparticles obtained *via* a very simple sol–gel synthesis method that can act as an excellent electrocatalyst. They show high electrocatalytic activity toward the reduction of H_2_O_2_ and achieve the highly sensitive detection of H_2_O_2_ with a small detection time and quick reproducibility for subsequent measurements. Table 1 summarizes research relating to non-enzymatic sensors for H_2_O_2_.

**Table 1:** A comparison of non-enzymatic electrochemical sensors for H_2_O_2_

Electrode	Linear range (mM)	LOD (μM)	Sensitivity (μA mM^−1^ cm^−2^)	Ref.
MnO_2_	0.00008–12.78	0.02	Not given	21
GO/MnO_2_	0.0049–4.5	0.48	191	22
MnO_2_/Nafion	0.001–0.015	2.0	400	23
Ag–MnO_2_ MWCNTs	0.005–10.4	1.7	82.5	24
MnO_2_/carbon fiber	0.012–0.26	5.4	10.6	25
MnO_2_ nanosheets/chitosan	0.005–3.5	1.5	130.56	26
MnO_2_ NPs-CNFs	0.01–15	1.1	71	27
MnO_2_–GO	0.005–0.6	0.8	38.2	28
Chromium(iii) hexacyanoferrate(ii)	0.01–1.3	0.03	0.1152	29
Chromium(iii) dicarboxylate MOF	25–500	3.52	11.9	30
MnO–CrN composite	0.0003–15	0.059	2156.25	This work

## Experimental

### Chemicals and reagents

All chemicals were purchased and utilized without any additional refinement.

### Synthesis of the MnO–CrN nanocomposite

In a typical synthesis method, Cr(NO_3_)_3_·9H_2_O and Mn(NO_3_)_2_·4H_2_O were dissolved at a 1 : 1 molar ratio in 30 ml of ethylene glycol under continuous stirring; the temperature was raised to 70 °C and maintained at this level for 1 h. After 1 h, we slowly added 2 M citric acid to the mixed metal solution under continuous stirring. The solution was maintained for a further 1 h and the temperature was raised to 180 °C. At this temperature, a sol–gel was obtained. To get the xerogel, it was put in an oven at 450 °C for 4 h. The material was ground using a pestle and mortar. Finally, the MnO–CrN composite was obtained *via* the ammonolysis of the xerogel at 800 °C for 8 h under NH_3(g)_ flow.

### Characterization techniques

The MnO–CrN composite synthesized after ammonolysis at 800 °C for 8 h under NH_3(g)_ flow was characterized using different techniques. The crystalline phase was studied using a MiniFlex 600 X-ray automated diffractometer using a monochromatic Cu-Kα source with *λ* = 0.1542 nm, *V* = 40 kV, and *I* = 15 mA. Phase identification information was collected over a 2*θ* angular range of 10–90° at a scanning rate of 1° min^−1^ to improve the accuracy. The morphology and microstructure were analyzed *via* scanning electron microscopy (SEM; JSM-7800F, Japan), operating at 15 kV, and transmission electron microscopy (TEM; FEI Tecnai F20) measurements. The pore-volume and surface-area measurements for the nitride material were performed applying Brunauer–Emmett–Teller (BET) theory and using an Accelerated Surface Area and Porosimetry system (ASAP 2420).

### Fabrication of the MnO–CrN modified electrode

Prior to the modification of the electrode, a mirror-smooth bare polished glassy carbon electrode (GCE) was obtained *via* using three different grades of alumina slurry: first 0.5 μm, then 0.1 μm, and finally 0.03 μm. The polished GCE was then washed using deionized water, ethanol, and deionized water again for 5 min consecutively. The GCE was dried using a nitrogen stream. The as-prepared MnO–CrN sample (5 mg) was uniformly dispersed in isopropanol : water (4 : 1) to make a homogenous ink. The MnO–CrN ink (3 μl) was dropped onto the surface of the GCE and was allowed to dehydrate at less than 60 °C in a drying oven for 10 min to obtain the MnO–CrN-modified electrode (MnO–CrN/GCE).

## Results and discussion

### XRD analysis

**Fig. 1:**
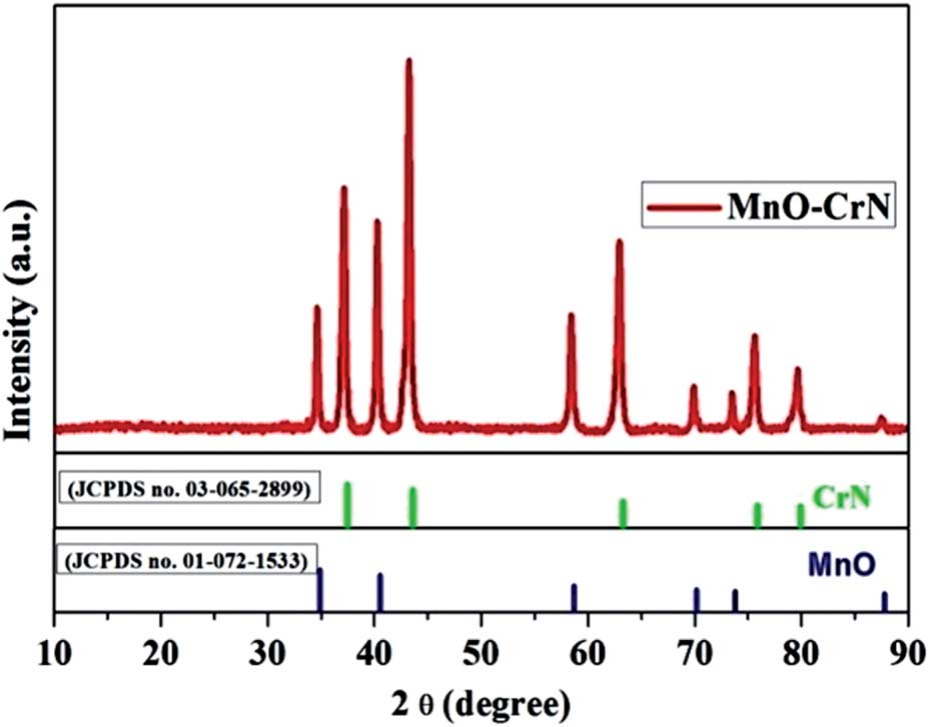
The XRD pattern of the MnO–CrN composite.

The crystallinity and phase formation of the prepared material was inspected *via* XRD. Fig. 1 shows the corresponding XRD pattern of the synthesized material, in which peaks emerged that can be indexed to the reflection planes of tetragonal α-MnO and cubic CrN. MnO, with the standard card number JCPDS-01-072-1533, is shown in blue, and CrN, matching the standard card number JCPDS-03-065-2899, is shown in green in Fig. 1. An additional important aspect is the morphological study and this was carried out *via* SEM and TEM analysis.

### Morphological characterization of the MnO–CrN nanocomposite

**Fig. 2:**
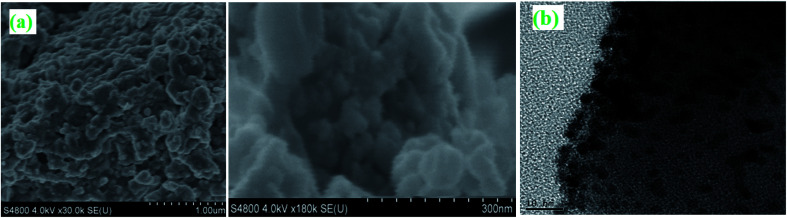
SEM (a) and TEM (b) images of the MnO–CrN composite.

Surface morphological studies of the MnO–CrN composite nanomaterial were done *via* SEM and TEM, as illustrated in Fig. 2. Fig. 2(a) illustrates the existence of flake-like particles with significantly varied sizes, and rigorously aggregated particles can be seen. For more structural detail, TEM analysis was carried out, which shows that the particles are in the nano-range and maybe this is the reason they are seen to aggregate in SEM analysis; on the other hand, in TEM analysis, it can be seen that the MnO–CrN composite particles are distributed homogeneously. They have a mean size of around 5–10 nm, increasing the specific surface area of the MnO–CrN composite. It should be noted that the performance of a sensor strongly depends on the existence of nano-size material particles.

### BET specific surface area analysis

**Fig. 3:**
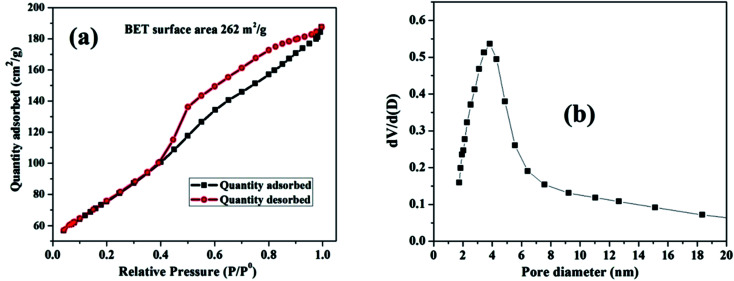
(a) The N_2_ adsorption–desorption isotherm for the MnO–CrN nanocomposite. (b) The corresponding BJH pore size distribution curve.

The pore size distribution and estimated surface area of the nanosized MnO–CrN composite were studied *via* nitrogen adsorption desorption isotherms of the as-synthesized material (Fig. 3). According to the IUPAC system of classification, the isotherms are type-IV.^31^ The specific surface area is found to be 262 m^2^ g^−1^.

The Barrett–Joyner–Halenda (BJH) method was applied to estimate the pore size diameter, as shown in Fig. 3(b), revealing the existence of mesopores in the composite with a diameter of around 4 nm. The synthesis of the nanosized mesoporous MnO–CrN composite will be very beneficial for sensing. Therefore, in the next step, we are going to study the application of the MnO–CrN composite to H_2_O_2_ sensing.

### Electrochemical measurements

All electrochemical measurements were performed using a CHI 760C workstation, which was utilized to control the applied voltage in a three-electrode system for amperometric analysis. First of all, 5 mg ml^−1^ material was dispersed in a 4 : 1 mixture of water and isopropanol with 20 μl of Nafion, using a sonication bath for 15 min. Ink was obtained after sonication, and 3 μl of this was cast onto a 2.5 mm polished GCE. 0.1 M PBS was used as a supporting electrolyte, and it was prepared *via* mixing 3.39 g of NaH_2_PO_4_ and 20.20 g of Na_2_HPO_4_ in 1 L of water. An Ag/AgCl (3 M KCl) electrode and platinum wire were employed as the reference and counter electrodes, respectively. The reduction and oxidation of H_2_O_2_ on the MnO–CrN electrode were quantified *via* CV in 0.1 M PBS buffer (pH 7.4). Before a certain amount of H_2_O_2_ was added, the buffer was subjected to de-oxygenation with pure nitrogen for 20 min. During calibration, the surface of the sample solution was gently purged with pure nitrogen gas to generate an inert atmosphere. The electrode was subjected to electrochemical treatment *via* cycling the potential from −1.0 to 1.2 V at 100 mV s^−1^ in 0.1 M PBS until stable voltammogram curves were obtained. The detection temperature of the electrochemical cell was room temperature, *i.e.*, 25 °C. Amperometric (*i*–*t*) analysis was carried out *via* the successive addition of H_2_O_2_ at −0.5 V *vs.* the Ag/AgCl electrode, obtained from CV curves.

### Cyclic voltammetry

**Fig. 4:**
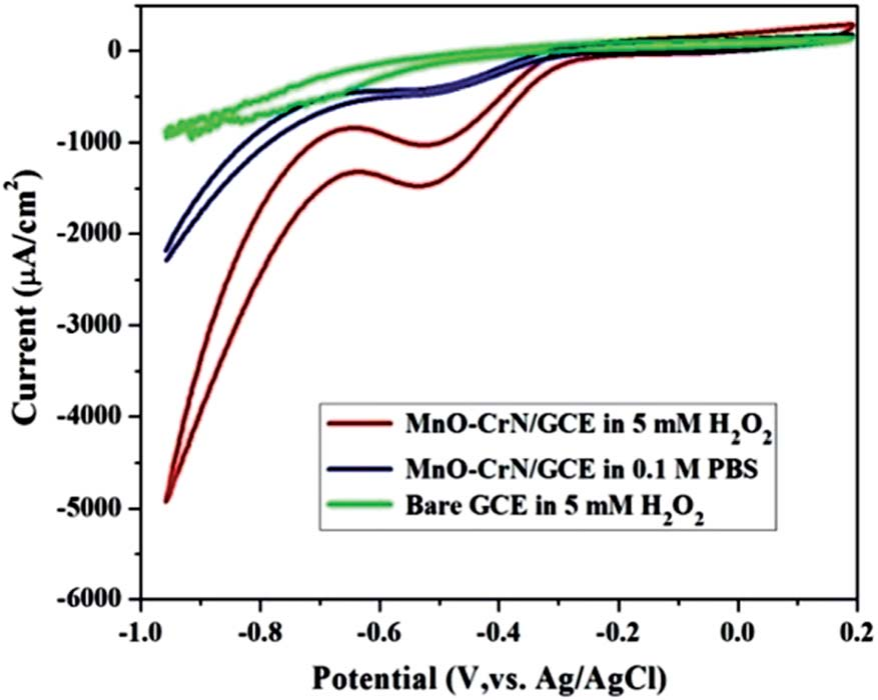
CV scans of bare and modified GCEs in a solution of 0.1 M PBS buffer (pH = 7.4) or 0.1 M PBS buffer (pH = 7.4) with 5 mM H_2_O_2_; scan rate: 100 mV s^−1^.

CV studies were carried out to analyze the electrocatalytic activity of the MnO–CrN composite material for the detection of H_2_O_2_. First, a bare GCE was tried for H_2_O_2_ detection in 5 mM H_2_O_2_ and negligible reduction behavior was observed. A modified glassy carbon electrode (MnO–CrN/GCE) was checked both in the presence and absence of H_2_O_2_ in 0.1 M PBS solution. A prominent reduction peak was observed when CV testing was carried out in 5 mM H_2_O_2_. Fig. 4 shows CV scans from all the above-mentioned circumstances. In the case of 5 mM (30 ml) H_2_O_2_ in 0.1 mol L^−1^ PBS with the modified MnO/CrN/GC electrode at 100 mV s^−1^ in a deoxygenated environment, a reductive current peak was seen in the range of −0.40 to −0.55 V, with the highest current obtained at −0.50 V. It is clear that the redox peaks could be assigned to electrochemical reactions involving MnO–CrN because no other interference peaks were observed from the background samples. These results indicate that the MnO–CrN composite showed excellent electrocatalytic performance for the reduction of H_2_O_2_ without being impeded in PBS matrix solution. The cathodic peak current, which is assigned to H_2_O_2_ reduction on the surface of the modified electrode, indicates that a controlled process happened during mass transfer on the electrode surface. It is clear that the reduction potential is due to MnO_2_ being combined with CrN. S. Lopa *et al.* studied pristine CrCl_3_ and a Cr^III/II^ MOF, which showed a lower potential (0.4 V) than MnO_2_.^30^ Therefore, CrN transfer electrons and acts as a backbone for MnO_2_.

### The amperometric response of the MnO–CrN electrode to H_2_O_2_

The performance of the synthesized MnO–CrN electrode towards H_2_O_2_ sensing was assessed through amperometric current–time (*i*–*t*) measurements after successively adding constant amounts of H_2_O_2_ into constantly stirred 0.1 M PBS solution (N_2_-saturated). Fig. 4 shows that the maximum current response toward the reduction of H_2_O_2_ was obtained at −0.50 V in the operating potential range of −0.45 to −0.55 V. Hence, −0.50 V was selected as the optimum reductive potential for the detection of H_2_O_2_ because the maximum reduction current is obtained at this potential.

**Fig. 5:**
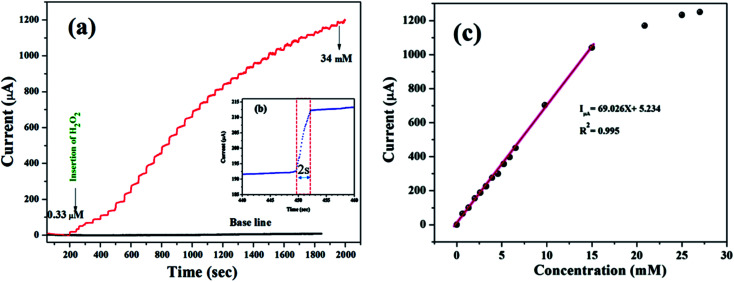
(a) The amperometric responses to the successive dropwise addition of H_2_O_2_ into 0.1 M PBS (pH = 7.4) at −0.50 V (*vs.* Ag/AgCl) under a continuously supplied N_2_ atmosphere. (b) The response time of the MnO–CrN-modified GCE electrode. (c) The respective calibration curve of H_2_O_2_ concentration *vs.* current.

According to the representative amperometric responses shown in Fig. 5(a), after the stepwise addition of H_2_O_2_ solution a well-ordered step-like response is seen swiftly, and a steady-state signal is attained within 2 s. The respective calibration curve for H_2_O_2_ detection is illustrated in Fig. 5(b). Firstly, H_2_O_2_ was adsorbed on the MnO–CrN surface, which was reduced to a lower oxidation state due to the absorbed H_2_O_2_; secondly, the lower oxidation state of MnO–CrN was oxidized and regenerated. Sensor calibration was executed three times and standard deviations were measured. The error bars of the mean current responses were obtained based on standard deviation; as the error bars cover a very small range in comparison to the mean values, they are barely noticeable in the plot. The response was quick and reached 95% of the steady-state value in less than 2 s, and the current rose linearly with the H_2_O_2_ concentration from 0.33 μM to 15 mM. The linear regression equation between 0.33 μM and 15 mM was determined to be: *I* = 69.02*x* + 5.234 (*R*^2^ = 0.995). The linear range of MnO–CrN/GCE for H_2_O_2_ amperometric detection was found to be from 0.33 μM to 15 mM in 30 ml of PBS (*R*^2^ = 0.995) with a limit of detection (LOD) of 0.059 μM and a limit of quantification of 0.199 μM (S/N = 3).

Based on linear calibration curves, the repeatability and accuracy depend on two important factors: the LOD and the LOQ (limit of quantification). Through such limits, it is possible to describe the smallest analyte concentration that can be consistently detected and quantified. The LOQ and LOD^32^ of MnO–CrN/GCE were calculated *via* applying eqn (1) and (2):1
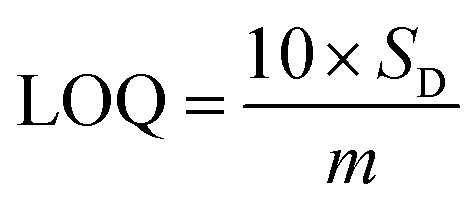
2
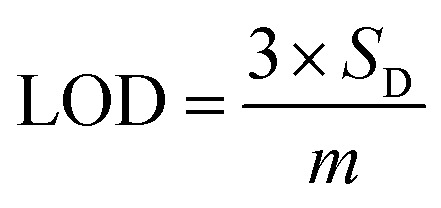
where *m* is the calibration curve slope, which is measured from the regression equation, and *S*_D_ is the standard deviation from the stable signal of blank solution. The electrode performance was superior compared to different previously reported Mn-based electrodes (Table 1). Consequently, the MnO–CrN-based modified electrode might be further used in sensors for the sensitive detection of H_2_O_2_ in 0.1 M PBS solution. It can be seen that the performance of the sensor is better than most other non-enzymatic H_2_O_2_ sensors when the MnO–CrN composite is used as an electrode. This might be associated with the higher specific surface area of the MnO–CrN composite, which offered a huge number of anchoring sites for MnO_2_ deposition during the preparation of the MnO–CrN composite and prohibited the aggregation of nanosized structures.

### Interference study

**Fig. 6:**
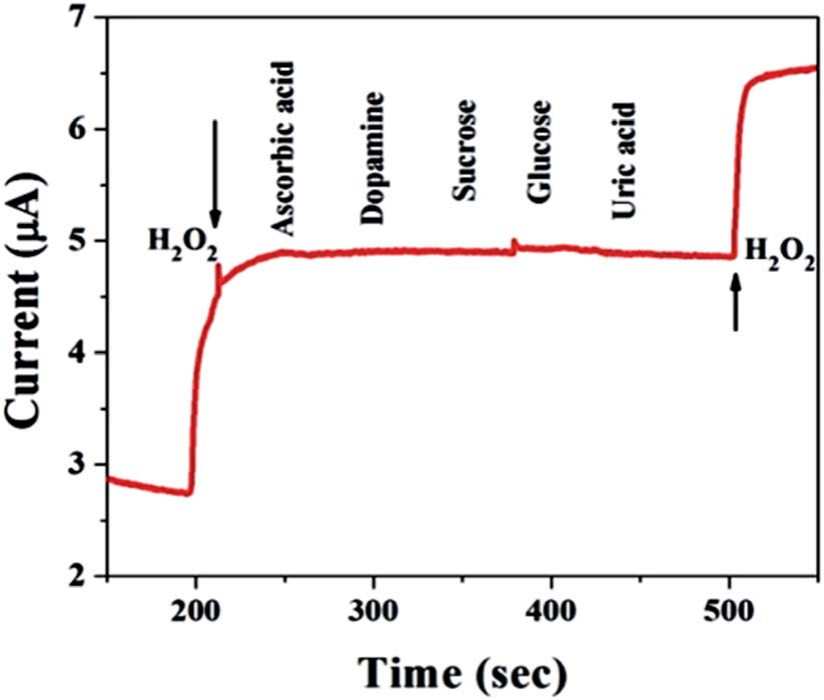
A study of the response of the MnO–CrN-modified GCE to interferents with the successive addition of 0.1 mM H_2_O_2_ and 1 mM interferents, *i.e.*, ascorbic acid, dopamine, sucrose, glucose, and uric acid, into continuously stirred N_2_-saturated 0.1 M PBS. Applied potential: −0.5 V; stirring rate: 400 rpm.

For practical applications, a critical property of H_2_O_2_ sensors is selectivity, which is evaluated here. Typical amperometric analysis of interference was carried out at a potential of 0.5 V *via* the successive injection of 0.1 mM H_2_O_2_ and 1 mM interferents, *i.e.*, ascorbic acid, dopamine, sucrose, glucose, and uric acid, into continuously stirred N_2_-saturated 0.1 M PBS (pH = 7.4). Fig. 6 shows a current response to H_2_O_2_, while the other interfering species do not result in a distinct current response, which suggests satisfactory selectivity for the electrochemical detection of H_2_O_2_ when using the MnO–CrN-modified GCE.

### Validation of the proposed sensor

In evaluating the analytical performance of a sensor, its shelf life, reproducibility, and enduring stability are imperative. Shelf-life testing was carried out *via* setting aside prepared MnO–CrN/GCE in air at room temperature, followed by measurements being conducted after four weeks. It was noteworthy that the sensitivity was long-lasting, for a period of four weeks. The repeatability of MnO–CrN/GCE has also been probed *via* observing the CV response toward 5 mM H_2_O_2_ for 20 cycles after 4 weeks (Fig. S1†). The induced current response from MnO–CrN/GCE remains similar and stable, with the retention of the initial current value, highlighting the good shelf life. The CrN/GCE electrode displays good electrocatalytic activity in relation to the reduction of H_2_O_2_ with a LOD of 0.059 μM toward H_2_O_2_ (S/N = 3).

To calculate the recovery abilities of the MnO–CrN-composite-based sensor, standard addition techniques were applied to detect H_2_O_2_ in defatted milk. Shop-bought defatted milk samples were used. We used three samples, and the study shows good reproducibility, in the range of 99.81–98.86% (Table 2).

**Table 2:** Recovery tests in defatted milk samples using the MnO–CrN-nanocomposite-based sensor

Sample	Added (v/v)	Found (mM l^−1^)	Recovery (%)
1	15	14.81	98.73
2	100	99.81	99.81
3	200	197.21	98.86

In order to determine the stability of the device, repeatability testing was carried out with a known amount of H_2_O_2_ in a defatted milk sample. Repeatability testing was carried out 100 times, as shown in Table 3, with a relative standard deviation (RSD) of 1.80% upon repeatedly measuring the H_2_O_2_ concentration in a milk sample. Hence, the sensor showed great sensitivity of 2156.25 μA mM^−1^ cm^−2^ with a wide linear range (0.33 μM to 15 mM), good selectivity for H_2_O_2_, and reproducibility in the range of 99.81–98.86% with a RSD of 1.81% and good stability.

**Table 3:** The repeatability of sample measurement with the MnO–CrN sensor

Sample	Number of measurements	Average value (mV)	SD (±)	RSD (%)
Defatted milk sample	100	1.385	0.025	1.80

## Conclusions

In summary, we disclosed for the first time the facile synthesis of a MnO–CrN composite material, and the subsequent manufacturing of an electrode made the current work of interest. The constructed synergism between MnO–CrN/GCE and H_2_O_2_ offers increased charge-transfer effectiveness. The unique features of fabricated MnO–CrN include its well-defined size and shape. A novel non-enzymatic H_2_O_2_ sensor based on MnO–CrN is productively developed, showing an outstanding electrochemical response with high accuracy and sensitivity, and satisfactory recovery. The novelty of this work is that we combined MnO with CrN as a conduction support material, rather than with a carbon-based material, and synthesized such a material for the first time at the nano-level *via* a new facile synthesis method. Here, CrN might be playing the role of a conducting material, which is the reason why the MnO–CrN composite is highly competent as a non-enzymatic electrochemical sensing material for H_2_O_2_. An assessment of the H_2_O_2_ sensing activity of MnO–CrN/GCE concludes that MnO–CrN/GCE has scope for future development due to its suitable LOD (0.059 μM) and sensitivity (2156.25 μA mM^−1^ cm^−2^).

## Conflicts of interest

All authors made equal contributions, and there are no conflicts of interest.

## Supplementary Material

RA-011-D1RA01485D-s001
