# Supplementary material for: A novel MnO–CrN nanocomposite based non-enzymatic hydrogen peroxide sensor
Source: RSC Adv. 2021 May 27;11(31):19316–22. doi: 10.1039/d1ra01485d (PMC9033555; doi:10.1039/d1ra01485d)
Supplement: RA-011-D1RA01485D-s001 [file RA-011-D1RA01485D-s001.pdf]

Electronic Supplementary Material (ESI) for RSC Advances.
This journal is © The Royal Society of Chemistry 2021

Supporting data

Novel MnO-CrN nanocomposite based non-enzymatic peroxide sensor

Ayesha Khan Tareen[1,2], Karim Khan[2,3,*], Waqas Ahmad [4], Muhammad Farooq Khan[5], Qudrat Ullah Khan, and Xinke liu[1,*].

1. *College of Materials Science and Engineering, Shenzhen University, Nanhai Ave 3688, Shenzhen, Guangdong 518060, P. R China.*
2. *Institute of Microscale Optoelectronics, Collaborative Innovation Centre for Optoelectronic Science & Technology, Key Laboratory of Optoelectronic Devices and Systems of Ministry of Education and Guangdong Province, College of Physics and Optoelectronic Engineering, Shenzhen Key Laboratory of Micro-Nano Photonic Information Technology, Guangdong Laboratory of Artificial Intelligence and Digital Economy (SZ), Shenzhen University, Shenzhen 518060, P.R. China.*
3. *School of Electrical Engineering & Intelligentization, Dongguan University of Technology, Dongguan (DGUT), Dongguan,523808, Guangdong Province, P. R. China.*
4. *International Collaborative Laboratory of 2D Materials for Optoelectronics Science and Technology of Ministry of Education, Institute of Microscale Optoelectronics, Shenzhen University, Shenzhen 518060, P. R. China.*
5. *Department of Electrical Engineering, Sejong University, 209 Neungdong-ro, Gwangjin-gu, 05006, South Korea.*
6. *College of physics and optoelectronics engineering, Shenzhen university.*

***Corresponding author: Karim Khan**: karim_khan_niazi@yahoo.com, and **Xinke liu**: xkliu@szu.edu.cn.

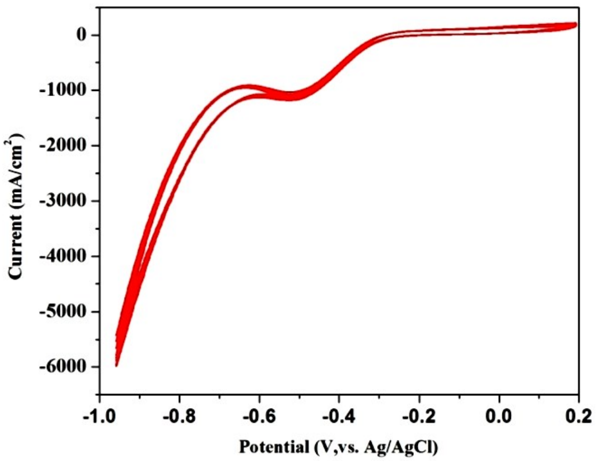

Fig.S1. The 20 CV cycles of modified MnO-CrN/GCE after four weeks in a solution of 5 mM H_2O_2. Scan rate: 100 mV/s.
